# Development and characterization of a synthetic DNA, NUversa, to be used as a standard in quantitative polymerase chain reactions for molecular pneumococcal serotyping

**DOI:** 10.1093/femsle/fnx173

**Published:** 2017-08-14

**Authors:** Fuminori Sakai, Griffin Sonaty, David Watson, Keith P. Klugman, Jorge E. Vidal

**Affiliations:** 1 Hubert Department of Global Health, Rollins School of Public Health, Emory University, 1518 Clifton Rd NE Room 6007, Atlanta, GA 30322, USA; 2 Bill and Melinda Gates Foundation, 500 Fifth Avenue North, Seattle, WA 98109, USA

**Keywords:** *Streptococcus pneumoniae*, serotype, qPCR, NUversa

## Abstract

Identification of *Streptococcus pneumoniae* and its more than 90 serotypes is routinely conducted by culture and Quellung reactions. Quantitative polymerase chain reactions (qPCRs) have been developed for molecular detection, including a pan-pneumococcus *lytA* assay, and assays targeting 79 serotypes. Reactions require genomic DNA from every target to prepare standards, which can be time consuming. In this study, we have developed a synthetic DNA molecule as a surrogate for genomic DNA and present new single-plex qPCR reactions to increase molecular detection to 94 pneumococcal serotypes. Specificity of these new reactions was confirmed with a limit of detection between 2 and 20 genome equivalents/reaction. A synthetic DNA (NUversa, ∼8.2 kb) was then engineered to contain all available qPCR targets for serotyping and *lytA*. NUversa was cloned into pUC57-Amp-modified to generate pNUversa (∼10.2 kb). Standards prepared from pNUversa and NUversa were compared against standards made out of genomic DNA. Linearity [NUversa (R^2^ > 0.982); pNUversa (R^2^ > 0.991)] and efficiency of qPCR reactions were similar to those utilizing chromosomal DNA (R^2^ > 0.981). Quantification with plasmid pNUversa was affected, however, whereas quantification with synthetic NUversa was comparable to that of genomic DNA. Therefore, NUversa may be utilized as DNA standard in single-plex assays of the currently known 94 pneumococcal serotypes.

## Teaser

NUversa is a synthetic DNA molecule that can be utilized as a ‘universal’ standard in virtually all serotype-specific qPCR assays for pneumococcus published in the literature.

## INTRODUCTION

*Streptococcus pneumoniae* (the pneumococcus) often causes life threatening infections, such as pneumonia, septicemia and meningitis (Klugman, Madhi and Albrich 2008; O’Brien *et al.*^2009^; van der Poll and Opal 2009). Pneumococcal disease (PD) kills ∼800 000 people, mostly children, every year worldwide (O’Brien *et al.*^2009^). To reduce the burden of PD, a 7-valent pneumococcal conjugate vaccine (PCV) was introduced in the USA in 2000 and was replaced by a 13-valent pneumococcal conjugate vaccine (PCV13) in 2010. Moreover, PCV has been introduced in many parts of the world including European countries. The introduction of these vaccines has reduced the burden of PD caused by vaccine serotypes on a global scale and has also decreased nasopharyngeal carriage of pneumococcal vaccine types in vaccinated populations (Simonsen *et al.*^2014^). There has been a modest increase of PD caused by non-vaccine types (NVT) since the introduction of PCV. In a phenomenon called serotype replacement, these strains have replaced vaccine-type (VT) strains in the nasopharynx, resulting in pneumococcal carriage rates similar to those observed prior to the introduction of vaccines (Singleton *et al.*^2007^; Weinberger, Malley and Lipsitch 2011; Feikin *et al.*^2013^).

The phenomenon of serotype replacement, and therefore the increase in prevalence of strains not included in current pneumococcal vaccines, might be of concern. NVT strains include more than 80 different serotypes. Monitoring the distribution of pneumococcal vaccine and non-vaccine serotypes is important for predicting the effectiveness of current vaccines, and might also be necessary for determining future vaccine formulations.

The Quellung reaction is the gold standard method for pneumococcal serotyping. Reactions utilize specific antibodies produced against the capsular polysaccharide (*cps*). In a positive reaction, antibodies produce ‘swelling’ of the pneumococcal capsule that can be observed under the microscope. While the Quellung reaction is proven, a number of molecular methods have been developed during last few years for molecular serotyping (Azzari *et al.*^2010^; Turner *et al.*^2011^; Azzari *et al.*^2012^; Pimenta *et al.*^2013^; Sakai *et al.*^2015^; Messaoudi *et al.*^2016^). Molecular methods are faster than the Quellung reactions, highly sensitive and specific, and adaptable to high-throughput platforms such as microarrays or TaqMan array cards (TACs). Satzke *et al.* recently evaluated molecular methods for molecular serotyping in a comprehensive, multi-center, comparative study (Satzke *et al.*^2015^). Real-time quantitative polymerase chain reaction (qPCR) proved to be highly specific and sensitive, with limits of detection (LOD) of reactions between ∼2 and ∼20 genome equivalents per reaction. qPCR reactions allow for the quantification of the bacterial load and can be utilized directly with DNA purified from nasopharyngeal specimens. The downside of qPCR reactions is that each reaction requires a specific DNA standard to construct the regression curve for the translation of real-time PCR results to genome equivalents; however, the exact whole genome size of many serotypes is frequently unavailable.

We, as well as others including the Centers for Disease Control and Prevention (CDC), have previously reported a series of serotype-specific single-plex and multiplex qPCR assays (Azzari *et al.*^2010^; Azzari *et al.*^2012^; Pimenta *et al.*^2013^; Sakai *et al.*^2015^; Messaoudi *et al.*^2016^; Pholwat *et al.*^2016^). Altogether, the reactions detect all 13 PCV types and 66 NVT strains, demonstrating high sensitivity and providing the absolute densities of individual serotypes in each sample. Although there is great progress with serotype coverage of qPCR reactions, there are still a number of serotypes for which molecular quantitative reactions are not available. In this study, we developed eleven novel single-plex qPCR assays for the detection and quantification of NVT strains. Furthermore, we engineered a synthetic DNA fragment containing sequences for *lytA* and for 94 pneumococcal serotypes, including targets for all qPCR assays developed thus far for pneumococcal serotyping. The synthetic DNA was cloned into a plasmid, hereafter called pNUversa, and was further characterized to be used as a standard for all qPCR reactions. Using pNUversa DNA as a standard allowed detection, but the plasmid's conformational structure affected quantification. A linearized pNUversa—a PCR product generated using pNUversa DNA—restored efficiency of quantitative reactions to levels similar to those achieved when using chromosomal DNA as a standard.

## METHODS

### Bacterial strains utilized in this study

Strains and reference strains utilized in this study were either originally isolated from clinical specimens and serotyped by Quellung reactions at Emory University, purchased from the Statens Serum Institut (Copenhagen, Denmark), or kindly provided by Dr Lesley McGee and Dr Bernard Beall from the CDC. Strains included serotypes: 1, 2, 3, 4, 5, 6A, 6B, 6C, 6D, 7A, 7B, 7C, 7F, 8, 9A, 9L, 9N, 9V, 10A, 10B, 10C, 10F, 11A, 11B, 11C, 11D, 11E, 11F, 12A, 12B, 12F, 13, 14, 15A, 15B, 15C, 15F, 16A, 16F, 17A, 17F, 18A, 18B, 18C, 18F, 19A, 19B, 19C, 19F, 19‘F’ (atypical), 20, 21, 22A, 22F, 23A, 23B, 23F, 24A, 24B, 24F, 25A, 25F, 27, 28A, 28F, 29, 31, 32A, 32F, 33A, 33B, 33C, 33D, 33F, 34, 35A, 35B, 35C, 35F, 36, 37, 38, 39, 40, 41A, 41F, 42, 43, 44, 45, 46, 47A, 47F and 48. To assess specificity, the following non-pneumococcal species found in the upper airways of healthy individuals were also included: *Streptococcus infantis*, *Streptococcus oralis*, *Streptococcus anguinosus*, *Streptococcus intermedius*, *Streptococcus sobrinus*, *Streptococcus pseudopneumoniae*, *Streptococcus mitis*, *Streptococcus parasanguinis*, *Streptococcus australis*, *Streptococcus mutans*, *Streptococcus peroris*, *Streptococcus oligofermentans*, *Streptococcus intestinalis*, *Streptococcus vestibularis*, *Streptococcus cristatus*, *Streptococcus salivarius*, *Streptococcus gordonii*, *Streptococcus sanguinis*, *Streptococcus sinensis* and *Dolosigranulum pigrum* (Carvalho Mda *et al.*^2012^). DNA from *Streptococcus pneumoniae* reference strain TIGR4 (GenBank accession no. Z_AAGY00000000) (Tettelin *et al.*^2001^) was utilized as the standard for the pan-pneumococcus *lytA* qPCR assay (Carvalho Mda *et al.*^2007^).

### Purification of DNA and preparation of DNA standards for validating qPCR assays

Strains were grown overnight on blood agar plates, and DNA was extracted using the QIAamp DNA Mini Kit (Qiagen, Hilden, Germany) as follows. Pneumococci were resuspended in tris-ethylenediaminetetraacetic acid (TE) buffer (10 mM tris-HCl, 1 mM ethylenediaminetetraacetic acid, pH 8.0) containing 40 mg/ml lysozyme and 75 U/ml mutanolysin (Sigma-Aldrich Co., Saint Louis, Missouri, USA). This suspension was incubated at 37°C for 1 h after which 20 μl of proteinase K were added and incubated for 30 min at 56°C. Then, 4 μl of RNase A (100 mg/ml) (Qiagen, Hilden, Germany) were added to the suspension and incubated 5 min at room temperature. The following steps were conducted as outlined by the manufacturer. The eluted DNA was quantified using the Nanodrop system (Nanodrop Technologies, Wilmington, Delaware, USA). DNA was diluted with TE buffer to obtain DNA standards of the following amounts per reaction: 1 ng, 100 pg, 10 pg, 1 pg, 100 fg, 50 fg and 5 fg. Considering the genome size of the reference strain TIGR4, 2.16 Mb (Tettelin *et al.*^2001^), the approximate genome equivalent for each DNA standard was: 4.29 × 10^5^, 4.29 × 10^4^, 4.29 × 10^3^, 4.29 × 10^2^, 4.29 × 10^1^, 2.14 × 10^1^ and 2.14 genome equivalents, respectively. The efficiency (i.e. regression curves) of reactions using the above standards was evaluated in each run. Primers and probes were optimized for each quantitative reaction. The LOD was determined from the lowest concentration standards that achieved a positive reaction in each assay. The Ct-value cut-off to distinguish a positive reaction from a negative reaction was 40.

### Development of novel qPCR assays for NVT *Streptococcus pneumoniae*

Novel qPCR assays for serotypes 10CF, 11BC, 16A, 17A, 17F, 19C, 24BF, 28AF, 32AF, 33C and 48 were developed. Systematic design of the new qPCR assays was essentially done as detailed in our previous study (Sakai *et al.*^2015^). Briefly, the pneumococcal *cps* locus was obtained from the GenBank Database. Accession numbers are listed in Table 1. Specificity *in silico* was analyzed using the National Center for Biotechnology Information-Basic Local Alignment Search Tool. Once specific sequences were selected within each locus, assays were designed utilizing software from Integrated DNA technologies (http://www.idtdna.com/site). All qPCR primers and probes were synthesized at Sigma-Aldrich Co. The assays were performed utilizing Platinum Quantitative PCR Super Mix-UDG (Life Technology, Carlsbad, California, USA) and 2.5 μl of pure DNA as template in a CFX96 real-time PCR-detection system (Bio-Rad, Hercules, California, USA).

**Table 1.** Novel quantitative PCR assays for non-vaccine serotypes.

Serotype(s)		Sequence^a^	Target region	Accession No.	Position	Size (bp)	Limit of detection genome equivalents (fg)	Concentration (nM)	Reaction efficiency (avg)
10CF	Forward	CGAGTTATGGATGTTCTTATTGGC	*wcjG*	CR931651	4808–4831	137	21.4 (50)	400	103.4
	Reverse	CCCAACCCCACTCTGTATTG			4925–4944			400	
	Probe	ACAGGGCAAGACTGTGAATATTGTTCCA			4837–4864			300	
11BC	Forward	TCAAATTTGGCGTATTGCTTATCA	*wzy*	CR931654	10 998–11 021	106	2.1 (5)	400	102.8
	Reverse	TGATTATGAGCATAGTTGATCCCC			11 103–11 126			400	
	Probe	TCCGTGGCAAGATTCTGGTGCTAAG			11 079–11 103			200	
16A	Forward	GCTAGCAGGAACTTTTCTAGGG	*wcxR*	CR931667	6675–6696	132	21.4 (50)	200	92.9
	Reverse	TCCCTGTCCAAATCCGAAAC			6787–6806			200	
	Probe	CCCACGGGATGAATCCATTATGGCG			6703–6727			200	
17A	Forward	TGATTATGTCATTCGATTGCTTGG	*wzy*	CR931669	13 895–13 918	112	2.1 (5)	400	93.5
	Reverse	AAATCCTAAAATTCCTGTTTGAAAAGC			13 980–14 006			400	
	Probe	ATTATGGGCGTGGGTTACCGTAGG			13 941–13 964			200	
17F	Forward	TGCTTTTGTGGGTAGGACAAG	*wzx*	CR931670	17 361–17 381	130	21.4 (50)	400	97.8
	Reverse	TTATCCCATAAACCTGAGGCG			17 470–17 490			400	
	Probe	TGCAGGTGATATGCGGAGCCAAT			17 440–17 462			200	
19C	Forward	AATGGTTTTCAGATTACTTGATAGCTC	*wchU*	CR931677	19 294–19 320	115	2.1 (5)	400	95.7
	Reverse	CGTTCCTTATGAGAGTGGTCAAG			19 386–19 408			400	
	Probe	TGTTCCTGCCCCCACATAATGAACT			19 343–19 367			200	
24BF	Forward	TCTGAAAGTAATTAGTAAGATTAACGGAAG	*wzy*	CR931688	15 038–15 067	133	2.1 (5)	400	101.5
	Reverse	TCCATCTACTTTTAAAATAGCTCCAAC			15 144–15 170			400	
	Probe	CCACAGTCCCAAAATTGTCAGCAACC			15 085–15 110			200	
28AF	Forward	CAACTACAGGTATTTTTGATATCGGAG	*wcxP*	CR931692	12 047–12 073	141	2.1 (5)	400	96.7
	Reverse	GTTTACTACGTTTGTGAAGCGC			12 166–12 187			400	
	Probe	AGAAAATAGTAGGTTGATTGGCGGTGCT			12 075–12 102			200	
32AF	Forward	GTACTTCCTGTTCTAGGCTTGG	*wzy*	CR931696	12 471–12 492	134	2.1 (5)	400	101.7
	Reverse	CCCAGAGGAAAATAGCGTCTC			12 584–12 604			400	
	Probe	TTGTTCAAACCCAACCACTGCTCC			12 492–12 515			200	
33C	Forward	CAGAGACAGTTTCAGCAAATCTTAG	*wzy*	CR931700	11 270–11 294	147	2.1 (5)	400	103
	Reverse	AGCCTACACCTCTTATAAACGTTG			11 393–11 416			400	
	Probe	CCGTGTCCTATCCACAAACTTGTCTTCC			11 327–11 354			200	
48	Forward	CAGGTTTTGCTTCATATGGGAG	*wzy*	CR931722	13 043–13 064	133	2.1 (5)	400	94.9
	Reverse	ATCGGCCAAAAGTTATCATTAGC			13 153–13 175			400	
	Probe	CGCTGCTTATGTGTATTACTCTCCCCTG			13 068–13 095			200	

a Probes were labeled at 5΄ with FAM (6-carboxyfluorescein) and at 3΄ with BHQ1 (Black Hole Quencher-1).

### Specificity of newly developed qPCR assays

Specificity was investigated as previously described (Sakai *et al.*^2015^). In short, two DNA libraries were prepared: one containing DNA from all known pneumococcal serotypes (N = 94) and a second library containing DNA from related bacterial species that inhabit the upper respiratory airways (N = 20). Purified DNA preparations were adjusted to 40 pg/μl, and 150 μl of each DNA was transferred to a 96-well microtiter plate and stored at –80°C until use. Specificity testing utilized 2.5 μl of DNA, equivalent to 100 pg (i.e. 4.29 × 10^4^ genome equivalents), which was transferred from the libraries to each reaction plate. Reactions were run in a CFX96 real-time PCR-detection system.

### Cloning and extraction of synthetic vector containing all qPCR target sequences for serotyping

**Figure 1.**
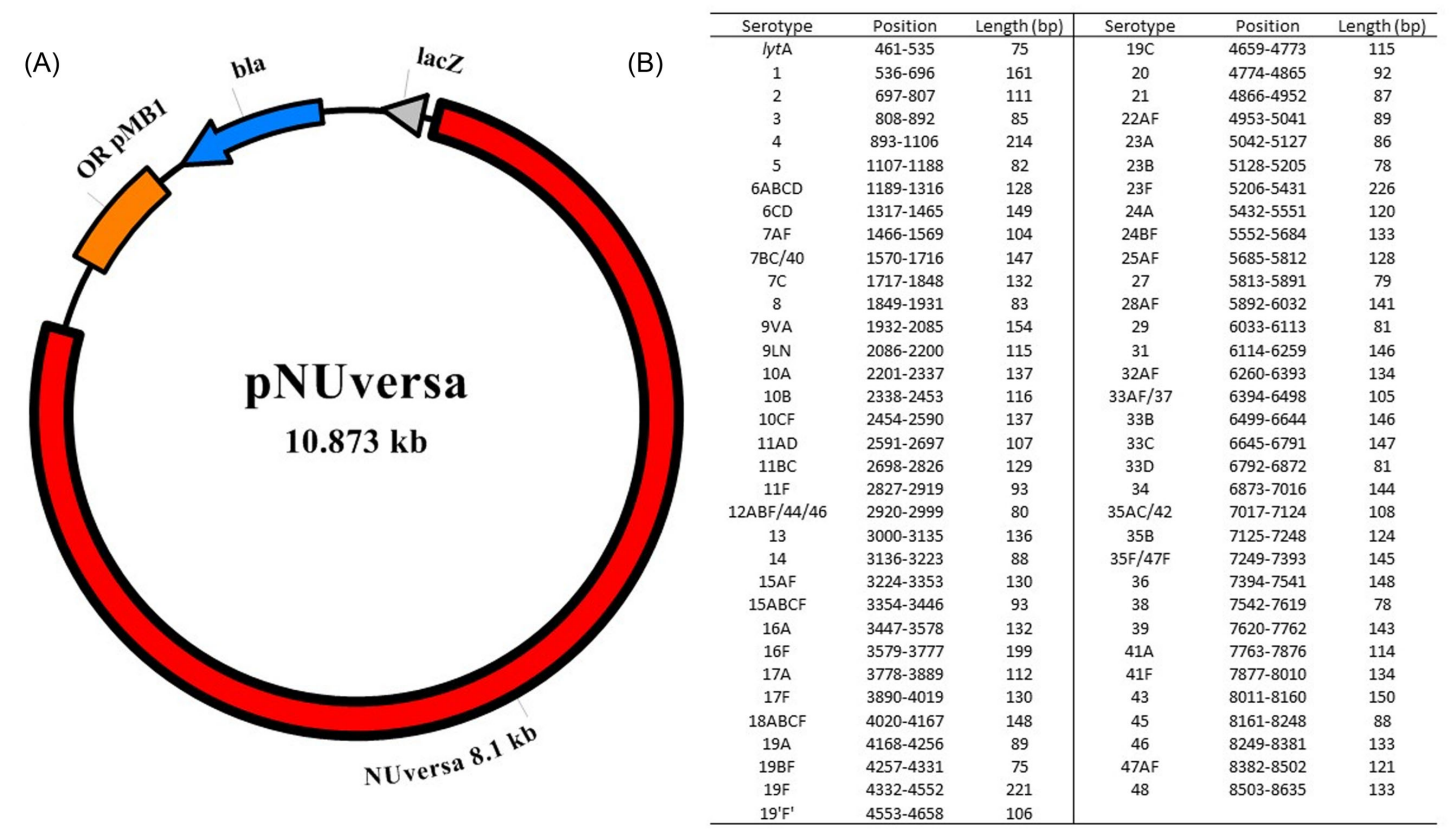
pNUversa plasmid map and position of sequences. (**A**) The synthetic NUversa gene (8175 bp) was cloned into a pUC57-Amp modified cloning vector to generate pNUversa (10 873 bp). The origin of replication (OR pMB1), ampicillin resistance gene (bla) and lacZ gene where the multiple cloning site is located are indicated. (**B**) Left panel shows the list of targets (N = 67) for single-plex quantitative PCR reactions included within NUversa. Targets for assays detecting more than one serotype, or serogroup, are denoted by a serogroup number and serotypes, i.e. 6ABCD, or serotypes separated by a slash, i.e. 7BC/40. Sequences are listed as they appear in pNUversa, including nucleotide position within NUversa and length of the fragment in bp.

The qPCR target sequences of all serotype-specific single-plex assays (N = 66), as well as *lytA*, were sequentially assembled into a single sequence using the DNASTAR Lasergene software version 11.2.1 (DNASTAR Inc., Madison, Wisconsin, USA). The final nucleotide sequence (∼8.2 kb) was synthesized and then subcloned within pUC57-Amp-modified (GenScript, Piscataway, New Jersey, USA). The synthetic vector (∼10.9 kb), named pNUversa (Fig. 1), was transformed into TOP10 competent *Escherichia coli* (Invitrogen, Carlsbad, California, USA) according to the manufacturer's instructions. pNUversa was purified from *E. coli* stocks using the QIAprep Spin Miniprep Kit (Qiagen, Hilden, Germany), eluted in EB buffer and quantified using the Nanodrop system. pNUversa was utilized as template in PCR reactions to produce the synthetic product, hereafter called NUversa.

### Long PCR and purification of the synthetic DNA (NUversa)

To produce the synthetic DNA fragment NUversa, long PCR was conducted with plasmid DNA from pNUversa as the template, platinum *Taq* DNA polymerase High Fidelity (Invitrogen, Carlsbad, California, USA) and the following primers whose complementary sequences are located upstream and downstream of NUversa: *lytA* re (5΄-TCGTGCGTTTTAATTCCAGCT-3΄: 200 nM) and Sero48 re (5΄-ATCGGCCAAAAGTTATCATTAGC-3΄: 200 nM). Reaction conditions were 30 s at 94°C for initial denaturation, followed by 35 cycles of 15 s at 94°C, 30 s at 60°C and 9 min at 68°C for amplification. PCR product was confirmed with electrophoresis in 1% agarose gel (Standard Low -m_r_ Agarose, Bio-Rad Laboratories Inc., Hercules, California, USA) with SYBR safe staining (Invitrogen, Carlsbad, California, USA). The obtained NUversa product was purified with the QIAquick PCR Purification Kit (Qiagen, Hilden, Germany) according to the manufacturer's instructions, eluted with EB buffer, evaluated for quality and quantified using the Nanodrop system. The purified NUversa product was stored at –80°C until use.

### Preparation of qPCR standards

To evaluate qPCR standards prepared with plasmid pNUversa DNA, and the synthetic NUversa PCR product, single-plex qPCR assays for the pan-pneumococcus *lytA* assay and serotype-specific qPCR assays targeting PCV13 serotypes were performed. Regression curves were obtained in each assay. Moreover, chromosomal DNA that was extracted from reference strains (TIGR4 and reference strains of serotype 1, 3, 4, 5, 6A, 7F, 9V, 14, 18C, 19A, 19F and 23F) was also included in these experiments. For quantification purposes, genome equivalents were estimated using the formula: molecules/mol × mol/g = molecules/g.

Utilizing Avogadro number and the average weight of a base pair (bp) [650 Da (g/mol)], the number of molecules were estimated as

^#^6.022 × 10^23^ (molecules/mole) × (gene amount (g)/(length of target gene (bp) × 650 (Da)).

^#^Avogadro number

Taking into account that the size of pNUversa and the NUversa PCR product were 10 873 bp and 8175 bp, respectively, genome equivalents contained in 100 pg and 10 pg of the vector and the PCR product were ∼8.52 × 10^6^ and ∼1.13 × 10^6^, respectively. DNA standards prepared by serial dilutions corresponded to [pNUversa] 8.52 × 10^6^, 8.52 × 10^5^, 8.52 × 10^4^, 8.52 × 10^3^, 8.52 × 10^2^, 8.52 × 10, 8.52 and 4.26, genome equivalents or [NUversa] 1.13 × 10^6^, 1.13 × 10^5^, 1.13 × 10^4^, 1.13 × 10^3^, 1.13 × 10^2^, 1.13 × 10 and 5.65, genome equivalents. Primer and probe sequences, and concentration, of VT single-plex qPCR assays were previously described (Pimenta *et al.*^2013^). qPCR assays were performed as mentioned above using Platinum Quantitative PCR SuperMix-UDG and the CFX96 real-time PCR-detection system.

## RESULTS

### Validation and optimization of quantitative PCR assays

Eleven newly designed qPCR assays (Table 1) were validated for their specificity using DNA from 94 pneumococcal serotypes and DNA from 20 non-pneumococcal species, most of which are inhabitants of the human upper airways, including several streptococcal species. Reactions were 100% specific as they only detected DNA in wells where the target pneumococcal serotype was added (not shown). Ct values of a typical positive reaction were between 20 and 30 cycles. Table 1 shows the optimal concentration that yielded a recommended reaction efficiency of between 90% and 110% and a correlation coefficient of nearly 1.000.

The LOD was also evaluated for each assay using an approach including serial dilution. Quantitative assays demonstrated an LOD of 5–50 fg per reaction, or ∼2–∼20 genome equivalents per reaction. Altogether, these new qPCR assays demonstrated high specificity, sufficient reaction efficiency to be utilized for quantification and detection of very low genome equivalents.

### Efficiency of quantitative reactions using NUversa is similar to those obtained using genomic DNA

We engineered a synthetic DNA molecule containing targets for most, if not all, qPCR reactions available in the literature (N = 66) including the *lytA* target and those sequences presented in this study that together target 94 pneumococcal serotypes. The synthetic DNA molecule was cloned into plasmid pUC57-Amp-modified and this new plasmid was named pNUversa (Fig. 1). The sequence of pNUversa was deposited in the GenBank with accession number MF540153. Purified pNUversa was then utilized as a template in qPCR reactions to generate linear DNA, called NUversa, which was used as a standard in *lytA*-based qPCR reactions.

**Figure 2.**
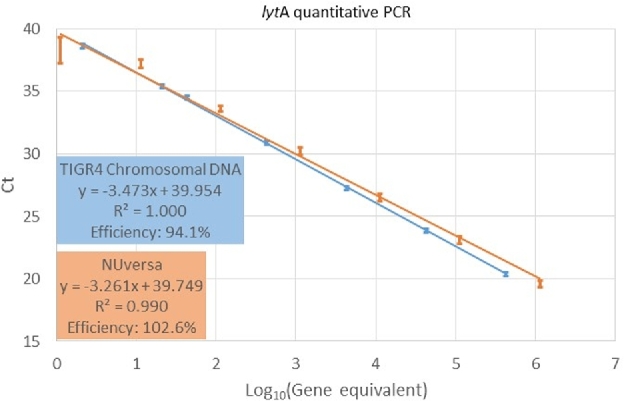
Linearity of qPCR reactions utilizing NUversa or genomic DNA. NUversa (orange) and genomic DNA (blue) were serially diluted to obtain seven standards (detailed in Methods) spanning 5 through 1.13 × 10^6^ and 2 through 4.29 × 10^5^ genome equivalents, respectively. Genome equivalent standards were utilized as template in qPCR reactions targeting the *lytA* gene. Plots represent the mean of cycles (Ct) of threshold values. Standard errors were calculated from four different replicates and each replicate included duplicate reactions. Regression equations (y), coefficient of determination (R^2^) and reaction efficiency are shown in the insets.

We first evaluated the quantitative nature of qPCR reactions targeting the *lytA* gene using NUversa DNA standards, and compared these results with those obtained utilizing the traditional standard genomic DNA from TIGR4. Efficiency of reactions from four independent experiments (i.e. each experiment performed in duplicate) with NUversa (94.1 ± 1.3) or genomic DNA (94.1 ± 3.2) fell within the recommended efficiency of >90% and <110% (Table 2). Regression curves showed a similar R^2^, 0.9993–1.000 when using TIGR4 genomic DNA and 0.991–0.997 when NUversa PCR product was utilized (Fig. 2). Standard deviations of Ct values obtained with the different standards ranged from 0.2555 to 0.3621 when utilizing TIGR4 genomic DNA, whereas those utilizing NUversa ranged from 0.4904 to 1.4602. Standard error of the means was 0.1278–0.1811 with TIGR4 genomic DNA and 0.2452–1.0325 with NUversa PCR product (Table 2). Further statistical analysis showed no significance when R^2^, the slope or y-intercept were compared by two-tailed *t*-Test (*P* > 0.05, all cases).

**Table 2.** *lyt*A quantitative PCR with TIGR4 genomic DNA and NUversa.

	Efficiency (%)	95.5	98.0	90.9	92.2			
Genomic DNA	Gen Eq^a^	Ct	Ct	Ct	Ct	Mean	SD	SEM
1 ng	4.29E+05	20.21	20.78	20.16	20.36	20.38	0.2819	0.1409
100 pg	4.29E+04	23.62	24.32	23.65	23.82	23.85	0.3215	0.1607
10 pg	4.29E+03	27.03	27.61	27.14	27.21	27.25	0.2555	0.1278
1000 fg	4.29E+02	30.52	31.25	30.73	30.94	30.86	0.3125	0.1563
100 fg	4.29E+01	34.47	34.36	34.22	34.90	34.49	0.2910	0.1455
50 fg	2.14E+01	34.90	35.35	35.59	35.73	35.39	0.3621	0.1811
5 fg	2.14E+00	38.20	38.68	38.98	38.60	38.61	0.3226	0.1613
NUversa	Efficiency (%)	92.8	93.3	94.7	95.8			
10 pg	1.13E+06	20.23	19.17	19.13	19.80	19.58	0.5336	0.2668
1000 fg	1.13E+05	23.89	22.60	22.73	23.27	23.12	0.5880	0.2940
100 fg	1.13E+04	27.37	26.02	26.23	26.57	26.55	0.5967	0.2983
10 fg	1.13E+03	31.08	29.50	30.02	30.23	30.21	0.6567	0.3283
1000 ag	1.13E+02	34.17	32.99	33.55	33.74	33.61	0.4904	0.2452
100 ag	1.13E+01	37.87	36.69	36.60	37.62	37.19	0.6455	0.3228
50 ag	5.65E+00	Negative	Negative	39.31	37.25	38.28	1.4602	1.0325

a Genome equivalents.

### The synthetic DNA (NUversa), but not the plasmid, can be utilized as standards for serotype-specific qPCR reactions

**Figure 3.**
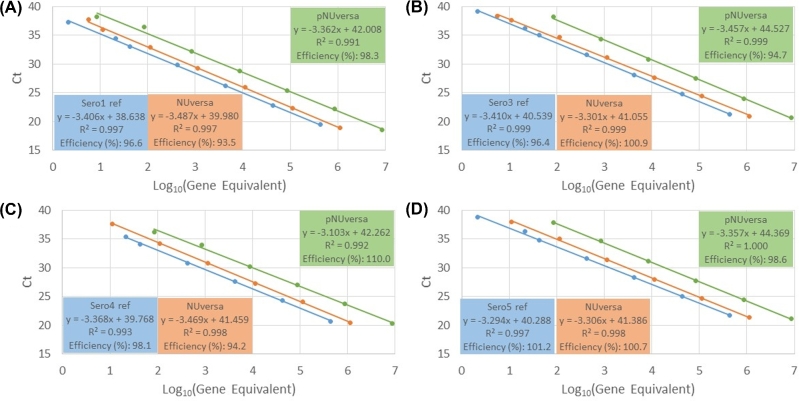
Linearity of qPCR reactions utilizing genomic DNA, NUversa or plasmid pNUversa. Genomic DNA (blue), NUversa (orange) or pNUversa (green), were serially diluted to obtain seven (genomic DNA and NUversa) or eight (pNUversa) standards (detailed in Methods) spanning 2 through 4.29 × 10^5^, 5 through 1.13 × 10^6^ and 4 through 8.52 × 10^6^ genome equivalents, respectively. Genome equivalent standards were utilized as template in serotype-specific qPCR reactions targeting (**A**) serotype 1, (**B**) serotype 3, (**C**) serotype 4 or (**D**) serotype 5. Plots represent the mean of cycles of threshold values obtained from duplicate reactions. Regression equations, coefficient of determination (R^2^) and reaction efficiency are shown in the insets.

Given that current real-time platforms are utilizing plasmid DNA as standards, we next investigated whether NUversa and pNUversa (i.e. the plasmid encoding NUversa) could be utilized as DNA standards for serotype-specific single-plex qPCR reactions. Serial dilutions of genomic DNA, pNUversa or NUversa were made and utilized as template in serotype-specific qPCR reactions targeting PCV13 serotypes, (i.e. 1, 3, 4, 5, 6A, 6B 7F, 9V, 14, 18C, 19A, 19F and 23F). Because most strains from where the genomic DNA was extracted have not been genome sequenced, the approximate genome equivalent was estimated using the whole genome size of serotype 4 strain TIGR4 (Tettelin *et al.*^2001^). As shown in Table 3, Fig. 3 and Supplementary Fig. S1, reaction efficiency of most qPCR assays using any of the standards fell within the recommended efficiency of >90% and <110%. The y-intercept, however, was only similar in reactions utilizing genomic DNA and NUversa, whereas reactions that utilized the plasmid pNUversa were statistically different to those with genomic DNA and those with NUversa (Table 3).

**Table 3.** Comparison of regression curve parameters obtained with serotype/serogroup-specific qPCR using genomic DNA, NUversa and pNUversa as standards.

						Serotype/serogroup-specific qPCR										
		1	3	4	5	6ABCD	7AF	9VA	14	18ABCF	19F	19A	23F	Average	SD	^b^t-Test
Efficiency (%)	^a^Chromosomal DNA	96.6	96.4	98.1	101.2	98.5	100.1	98.2	91.2	99.0	101.1	91.6	96.0	97.3	3.247	
	NUversa linear	93.5	100.9	94.2	100.7	91.7	94.1	107.5	100.0	95.7	95.6	94.8	95.8	97.0	4.417	0.859
	pNUversa	98.3	94.7	110.0	98.6	97.1	96.0	101.5	94.6	106.4	96.8	90.9	100.5	98.8	5.268	0.327
The coefficient of	^a^Chromosomal DNA	0.997	0.999	0.993	0.997	0.999	0.997	0.992	0.994	0.994	0.982	0.981	0.999	0.994	0.006	
determination (R^2^)	NUversa linear	0.997	0.999	0.998	0.998	0.990	0.993	0.987	0.982	0.966	0.997	0.988	0.999	0.991	0.010	0.438
	pNUversa	0.991	0.999	0.992	1.000	0.994	0.992	0.999	0.997	0.995	0.997	0.995	0.999	0.996	0.003	0.298
Slope	^a^Chromosomal DNA	–3.406	–3.410	–3.368	–3.294	–3.357	–3.319	–3.366	–3.554	–3.347	–3.295	–3.540	–3.422	–3.390	0.084	
	NUversa linear	–3.487	–3.301	–3.469	–3.306	–3.539	–3.471	–3.154	–3.321	–3.429	–3.432	–3.452	–3.427	–3.399	0.108	0.825
	pNUversa	–3.362	–3.457	–3.103	–3.357	–3.393	–3.422	–3.287	–3.458	–3.178	–3.402	–3.562	–3.310	–3.358	0.126	0.353
y-intercept	^a^Chromosomal DNA	38.638	40.539	39.768	40.288	40.793	39.601	40.084	38.990	38.959	39.667	40.468	40.245	39.837	0.691	
	NUversa linear	39.980	41.055	41.459	41.386	41.912	40.813	39.744	38.545	39.631	38.976	40.323	39.795	40.302	1.040	0.081
	pNUversa	42.008	44.527	42.262	44.369	43.615	43.144	42.894	41.956	41.524	41.889	44.880	42.240	42.942	1.157	2.49E-08

a Serotypes of strains for chromosomal DNA preps were 1, 3, 4, 5, 6A, 7F, 9V, 14, 18C, 19F, 19A and 23F

b Set of parameter values from NUversa and pNUversa reactions were statistically compared to values from chromosomal DNA reaction with unpaired, two-tailed t-test.

## DISCUSSION

In this study, NUversa, a synthetic DNA molecule was thoroughly characterized for its use as a standard in quantitative single-plex reactions for PCV13 pneumococcal serotypes including the pan-pneumococcus *lytA* assay. NUversa has the potential to be utilized in reactions targeting most pneumococcal serotypes. We also developed, tested and optimized eleven new real-time qPCR assays for the detection and quantification of 16 pneumococcal serotypes/serogroups belonging to NVT. While these NVT strains are less likely to be isolated from cases of PD, the emergence of NVT due to serotype replacement after vaccine introduction warrants development of assays for the detection of potential emergent strains and their use in epidemiological surveillance programs (Wyres *et al.*^2013^; Mosser *et al.*^2014^; van der Linden *et al.*^2015^).

To the best of our knowledge, many of the molecular assays (i.e. conventional PCR, qPCR, etc.) for the detection of target pneumococcal serotypes that were presented in this study have not previously been available. These new reactions, along with other qPCR reactions published by other groups, including the CDC, complete the coverage of the 94 pneumococcal serotypes for which an antiserum is available. We acknowledge that single-plex reactions for pneumococcal serotyping have a limited use in epidemiological studies because of the associated cost, need for increased amounts of DNA sample and increased time required to perform single-plex assays. However, validation studies conducted in this manuscript will allow qPCR assays, primers and probes described here to be incorporated into high-throughput technology such as multiplex qPCR schemes (Pimenta *et al.*^2013^) or a recently developed TAC for pneumococcal serotyping (Pholwat *et al.*^2016^). The TAC, for example, detects 78 different pneumococcal serotypes. It utilizes either DNA from *Streptococcus pneumoniae* isolates or DNA purified from clinical specimens as a template. This method yields fast and comprehensive serotyping in comparison to the standard method of Quellung reactions, but currently misses detection of >10 pneumococcal serotypes. The new reactions developed and validated here can be added to the TAC method, which will increase the number of molecular targets to 94 pneumococcal serotypes.

Another contribution of the studies presented within this manuscript was the characterization of NUversa and pNUversa. NUversa includes sequences from 67 qPCR targets that together detect 94 serotypes and performs as well as genomic DNA when utilized as a DNA standard in qPCR *lytA*-based reactions. This synthetic DNA can be utilized as a positive control or as a DNA standard for quantitative reactions in any laboratory where single-plex qPCR is performed. Moreover, with further optimization, NUversa could also be used as a control in multiplex reactions. Supporting this, we tested NUversa in two different multiplex reactions published by the CDC (Pimenta *et al.*^2013^), and NUversa was detected by all reactions within these multiplex reactions (not shown).

Using NUversa will allow a faster turnaround of data and findings, and it will be particularly helpful in resource limited settings, where maintaining a strain library required for current methods is prohibitively expensive and time consuming. To calculate genome equivalents per mass units (i.e. *in silico*), the genome size of the strain utilized to prepare DNA standards is required. A reference, genome-sequenced, TIGR4 strain has been classically utilized to prepare standards for quantitative reactions targeting the pan-pneumococcus *lytA* qPCR assay (Tettelin *et al.*^2001^; Carvalho Mda *et al.*^2007^). This strain has been widely distributed among research laboratories, and it is accessible to clinical laboratories. However, widely accessible pneumococcal strains belonging to all known serotypes to be utilized as a standard for serotype-specific single-plex qPCR reactions represent a challenge. Given that NUversa also contains the target for the pan-pneumococcus *lytA* assay, quantification using NUversa will be standardized in all studies, avoiding the use of subjective quantitative numbers obtained from DNA of poorly characterized strains as a DNA standard for constructing the standard curve. pNUversa, the plasmid encoding the NUversa synthetic sequence, was designed in a manner that allows for more sequences to be cloned into it if more serotypes are discovered in the years to come, or if new improved reactions, i.e. targets, become available for the current serotypes.

Different multiplex molecular platforms for the detection of human pathogens have been developed during the last few years (Kodani and Winchell 2012; Pholwat *et al.*^2015^). These platforms commonly utilize a plasmid containing targets for reactions detected by the systems. According to Hou *et al.*, however, a circular plasmid is not recommended as a quantification standard for real-time PCR assays because it frequently forms supercoiled structures; this unknown conformational structure may restrict polymerase reaction and cause delayed Ct of corresponding standards (Hou *et al.*^2010^).

In the current study, detection of target sequences in a plasmid (pNUversa) did not pose major issues but the quantitative nature of this system was proven to be inaccurate and required a synthetic linear DNA molecule. Absolute quantitative assays for PCV13 serotypes were conducted to compare the reactivity of chromosomal DNA and two types of synthetic genes: a circular plasmid and NUversa PCR product. Whereas the regression curves generated from chromosomal DNA were highly similar to those from NUversa PCR product, a significant discrepancy was found between the y-intercepts of the chromosomal DNA and the y-intercept of circular pNUversa reactions. These high y-intercept values with pNUversa caused apparent overestimation of quantification values when compared to both chromosomal DNA and NUversa. Using a plasmid as standard for quantification purposes, at least in this case, overestimates the quantity of corresponding Ct values. Together, these data indicate that synthetic NUversa is as efficient as genomic DNA to quantify genome equivalents in serotype-specific qPCR reactions. We, however, did not perform qPCR assays for the remaining NVT that can be a limitation of our study. Another limitation includes the diversity of normal flora strains utilized for the validation studies. Whereas specificity was investigated using DNA from all 94 serotypes, 20 different normal flora streptococci and other respiratory bacteria, we did not include all potential bacterial species that can colonize the human upper airways.

In this study, the whole genome size of serotype reference strains was estimated utilizing the genome size of TIGR4. However, this estimation may cause inaccurate quantification since the whole genome size might vary between serotypes. For further assessment of this synthetic DNA, *lytA* qPCR assays with TIGR4 chromosomal DNA and NUversa PCR product were compared. Since the genome sizes of both DNA preparations were available, specific genome equivalents for each standard could be estimated. The obtained regression curves from these two sources of standards were highly similar and no significant (i.e. statistically) discrepancy was observed. This result emphasized the significance of linearity and accurate gene size for absolute quantification, and demonstrated that NUversa PCR product can be utilized instead of chromosomal DNA in the assay (Hou *et al.*^2010^).

In summary, the synthetic NUversa DNA and the new single-plex reactions included in this study will be useful to many in the field conducting surveillance of the pneumococcus and pneumococcal serotypes by qPCR. Integrating NUversa and these qPCR assays with other previously reported high-throughput systems, such as the TAC system, will improve investigations of disease trends in this age of serotype replacement post-vaccination. These advancements will greatly contribute to the ongoing monitoring and evaluation of pneumococcal immunization programs around the world.

## Supplementary Material

Supplemental material Supplementary data are available at *FEMSLE* online.
