# Supplementary material for: Development and characterization of a synthetic DNA, NUversa, to be used as a standard in quantitative polymerase chain reactions for molecular pneumococcal serotyping
Source: FEMS Microbiol Lett. 2017 Aug 14;364(17):fnx173. doi: 10.1093/femsle/fnx173 (PMC5812490; doi:10.1093/femsle/fnx173)
Supplement: Supplemental material — Supplementary data are available at FEMSLE online. [file fnx173_supp.zip › Supplimentary Figure 1 legend.docx]

**Supplementary Figure 1. Legend**

Linearity of qPCR reactions utilizing NUversa, plasmid pNUversa, or genomic DNA. NUversa (orange), pNUversa (green) or genomic DNA (blue), was serially diluted to obtain seven genome equivalents (detailed in Material and Methods) spanning 2 through 2x10^5^ genome equivalents. Genome equivalent standards were utilized as template in qPCR reactions targeting (A) serotype 6ABCD, (B) serotype 7AF, (C) serotype 9AV, (D) serotype 14, (E) serotype 18ABCF, (F) serotype 19A, (G) serotype 19F and (H) serotype 23F. Plots represent the mean of cycles of threshold values obtained from duplicate reactions. Regression equations, coefficient of determination (R^2^) and reaction efficiency are shown in the insets.
