# Supplementary figures and images for: Development and characterization of a synthetic DNA, NUversa, to be used as a standard in quantitative polymerase chain reactions for molecular pneumococcal serotyping
Source: FEMS Microbiol Lett. 2017 Aug 14;364(17):fnx173. doi: 10.1093/femsle/fnx173 (PMC5812490; doi:10.1093/femsle/fnx173)

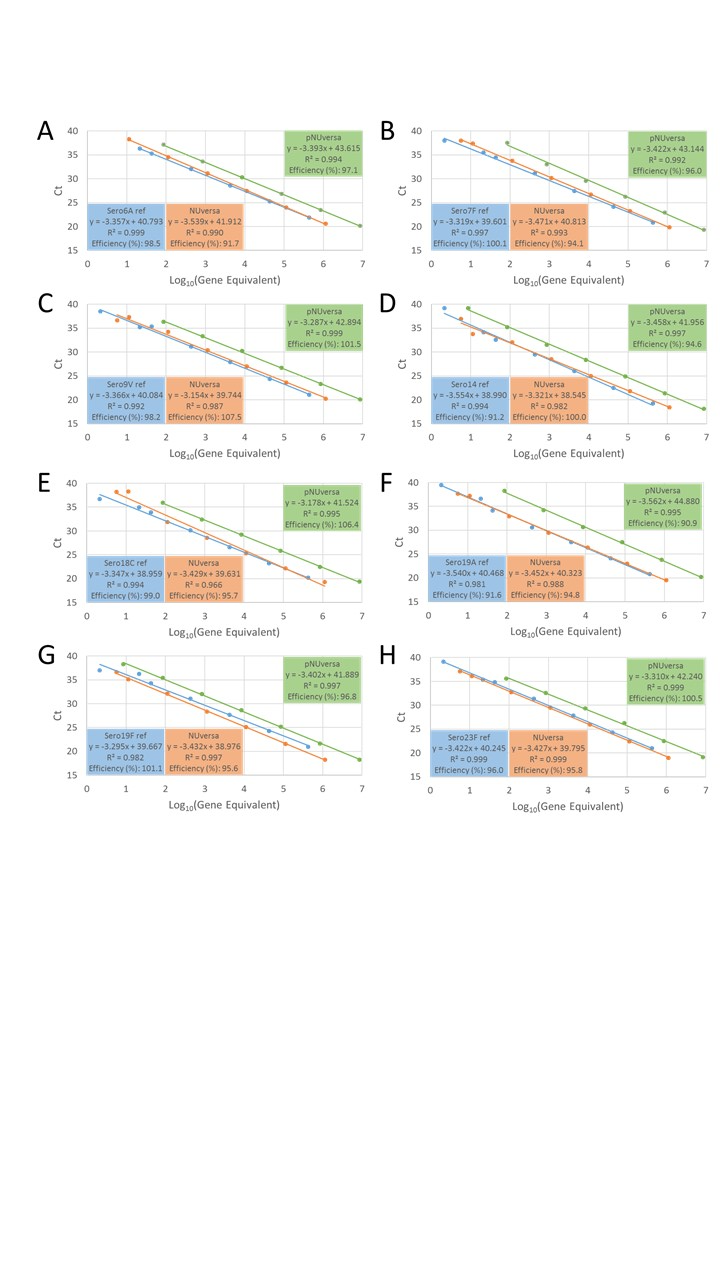

Supplement: Supplemental material — Supplementary data are available at FEMSLE online. [file fnx173_supp.zip › Supplementary Figure 1.png]
